# Antioxidant activity and free radical-scavenging capacity of *Gynura divaricata* leaf extracts at different temperatures

**DOI:** 10.4103/0973-1296.75900

**Published:** 2011

**Authors:** Chunpeng Wan, Yanying Yu, Shouran Zhou, Wei Liu, Shuge Tian, Shuwen Cao

**Affiliations:** 1 *State Key Laboratory of Food Science & Technology, Nanchang University, Nanchang - 330 047, Jiangxi,, China*; 2 *Department of chemistry, Nanchang University, Nanchang - 330 031, Jiangxi, China*; 3 *Jiangxi University of Traditional Chinese Medicine,, Nanchang - 330 006, Jiangxi, China*; 4 *Xinjiang Key Laboratory of Famous Prescription and Science of Formulas, Urumqi - 830 011, Xinjiang, China*

**Keywords:** Antioxidant activity, extraction temperature, *Gynura divaricata*, total phenolic content, total flavonoid content

## Abstract

**Background:**

Extraction temperature influences the total phenolic content (TPC), total flavonoid content (TFC) of medicinal plant extracts to a great extend. TPC and TFC are the principle activity constituents present in the plant. The effects of extraction temperature on TPC, TFC and free radical-scavenging capacity of *Gynura divaricata* leaf extracts are worth to study.

**Materials and Methods:**

Folin–Ciocalteu and aluminum chloride colorimetric assay were used to determine the TPC and TFC of *Gynura divaricata* leaf extracts at different temperatures. The antioxidant and free radical-scavenging activity were measured by 1,1-diphenyl-2-picrylhydrazyl (DPPH), 2,2-azino-bis-(3-ethylbenzthiazoline-6-sulfonic acid (ABTS) and phosphomolybdenum methods.

**Results:**

TPC and TFC were significantly elevated with increasing extraction temperature (from 40°C to 100°C). However, TPC and TFC were not significantly different (P > 0.05) at the extraction temperatures 90°C and 100°C. Also, the extracts obtained at a higher temperature exhibited a significant free radical-scavenging activity compared with extraction at lower temperatures (*P* < 0.05). The TPCs (13.95-36.68 mg gallic acid equivalent/g dry material) were highly correlated with DPPH (*R*^2^ = 0.9229), ABTS (*R*^2^ = 0.9951) free radical-scavenging capacity, and total antioxidant activity (*R*^2^ = 0.9872) evaluated by phosphomolybdenum method.

**Conclusion:**

The TPC and TFC of *G. divaricata* leaf was significantly influenced by the extraction temperatures, which were the main antioxidant constituents present in the *G. divaricata* plant.

## INTRODUCTION

Reactive free radicals, including superoxide, hydroxyl radical, and peroxyl radical, generally result in degradation of protein, lipid peroxidation, and oxidation of DNA, which have been considered to be linked with many chronic diseases, such as diabetes, cancers, and atherosclerosis.[1,2] Polyphenols are the major antioxidant constituents isolated from many medicinal and edible plants.[3] The extraction process does affect the yield, and to some extent, affects the stability of polyphenols.[4,5]

*Gynura divaricata* DC. (Compositae) is a traditional Chinese medicinal plant, commonly used for the treatment of bronchitis, pulmonary tuberculosis, pertussis, sore eye, toothache, rheumatic arthralgia, and diabetes in folk medicine.[6] The ethanol extract of *G. divaricata* has hypoglycemic activity in animal models.[7,8] It has also been reported that many constituents with antiproliferation activity exist in *G. divaricata*.[9,10] The chemical constituents of *G. divaricata* leaves include flavonoids, phenolics, cerebrosides, polysaccharide, alkaloids, terpenoids, and sterols.[9–12] Phenolics and flavonoids are the major antioxidant components of *Gynura procumbens* leaves. A previous report indicated that the concentration of phenolics in the extract is significantly influenced by the extraction temperature; that is, increasing the extraction temperature will decrease the total phenolic content (TPC).[13] However, no literature data are available on the effect of extraction temperature on the TPC, total flavonoid content (TFC), and free radical-scavenging capacity of *G. divaricata* leaves. Recently, we used single-factor experiments to optimize the extraction conditions of TPC from *G. divaricata*. An interesting phenomenon was found where the TPC was significantly elevated with increasing the extraction temperature, which was quite different from the same genus plants described in the literature.[13] The present study aims to evaluate the effect of extraction temperature on TPC, TFC, and free radical-scavenging capacity of *G. divaricata* leaves.

## MATERIALS AND METHODS

### Chemicals and reagents

Gallic acid (GA), trolox, quercetin, and 1,1-diphenyl-2-picrylhydrazyl (DPPH), and Folin–Ciocalteu’s phenol reagent were purchased from Sigma Chemical Company (St. Louis, MO, USA) and 2,2-azino-bis-(3-ethylbenzthiazoline-6-sulfonic acid) (ABTS) was obtained from TCI-SU (Tokyo, Japan). Potassium persulfate, aluminum chloride, sodium acetate, and all solvents used were of analytical grade and purchased from Sinopharm Chemical Reagent Co., Ltd (Shanghai, China). Visible spectra measurements were done using UV-2450 spectrophotometer (Shimadzu, Japan).

### Plant material

The *G. divaricata* was obtained in 2009 from Guangdong Province, China. A voucher specimen (201001) was deposited at the Department of Chemistry, Nanchang University. The leaves of *G. divaricata* were dried at 40°C in a hot air oven and finely powdered.

### Extraction

Samples of the powered leaf (1 g) were weighed and transferred into conical flasks. The samples were refluxed and extracted for 30 min with 50 mL of 45% aqueous ethanol at different temperatures (40°C, 60°C, 80°C, 90°C, 100°C). Single-factor experiments demonstrated that the 45% aqueous ethanol and 30 min extraction were the optimal extraction conditions (data not shown). The extracts were centrifuged (10000 *g*, Beckman, USA) and the supernatant was adjusted to a final volume of 50 mL with 45% aqueous ethanol.

### Determination of total phenolic and flavonoid content

The TPC in the extracts was determined by using Folin–Ciocalteu’s phenol reagent and external calibration with GA and the results were expressed as mg GA/g dry material.[14] Briefly, 0.2 mL of extract solution in a test tube and 5 mL of Folin–Ciocalteu’s phenol reagent (diluted 10 times) were added and the contents mixed thoroughly. After 4 min, 4 mL of sodium carbonate (7.5% w/v) was added to the mixture. The mixed solution was then immediately diluted to required volume (25 mL) with deionized distilled water and mixed thoroughly. The tubes were allowed to stand for 90 min before absorbance at 760 nm was measured by using UV-2450 spectrophotometer (Shimadzu, Japan). The TPC were calculated by using GA calibration curve. The calibration equation for GA was *Y* = 0.07411*X* + 0.0589 (*R*^2^ = 0.9977).

The TFC in the extracts were determined by using the colorimetric assay with slight modifications.[15] Briefly, 0.3 mL solution of extracts in 45% ethanol were separately mixed with 8 mL of 10% aluminum chloride, 4 mL of 0.2 M sodium acetate. The mixed solution was then immediately diluted to volume (25 mL) with deionized distilled water and mixed thoroughly and left at room temperature for 30 min. The absorbance of the reaction mixture was measured at 350 nm by using UV-2450 spectrophotometer (Shimadzu, Japan). TFCs were calculated by using kaempferol calibration curve. The calibration equation for kaempferol was *Y* = 0.04177*X* + 0.014181 (*R*^2^ = 0.9993). The results were expressed as mg kaempferol/g dry material.

### DPPH free radical-scavenging capacity

The free radical-scavenging activity of the extracts was evaluated by 1,1,-diphenyl-2-picryl-hydrazil (DPPH) using the method of Sajjad and Patel[16,17] with slight modifications. Briefly, DPPH solution (0.6 mM) was prepared in ethanol and 0.5 mL of this solution was mixed with 0.5 mL of 10-fold diluted extracts (final concentration 0.20 mg/mL dry material). The volume of the solution was adjusted with ethanol to a final volume of 5 mL. After incubation in a dark place for 30 min at room temperature, the absorbance of the mixture was measured at 515 nm against ethanol as blank using UV-2450 spectrophotometer (Shimadzu, Japan). Trolox (0.15 mg/mL) and Quercetin (0.1 mg/mL) were used as positive controls. The activities of the samples were evaluated by comparison with a control (containing 0.5 mL of DPPH solution and 4.5 mL of ethanol). Each sample was measured in triplicate and averaged. The activity was calculated according to the following formula:

$$DPPH\;scavenging\;activity\;(\%)\;=\;\left[\left(A_{c}–A_{s}\right)/A_{c}\right]\;\times \;100$$

where *A*_C_ is the absorbance value of the control and *A*_S_ is the absorbance value of the added test samples solution.

### ABTS cation free radical-scavenging activity

For ABTS assay, the procedure followed was the method of Dimitrina and Roberta[18,19] with some modifications. ABTS was dissolved in water to make a concentration of 7 mmol/L. ABTS ^+^ was produced by reacting the ABTS stock solution with 2.45 mmol/L potassium persulfate (final concentration) and allowing the mixture to stand in the dark at room temperature for 12–16 h before use. For the test of samples, the ABTS^+^ stock solution was diluted with 80% methanol to an absorbance of 0.70 ± 0.02 at 734 nm. After the addition of 4.85 mL of diluted ABTS ^+^ to 0.15 mL of 10-fold diluted samples (final concentration 0.06 mg/mL dry material), the absorbance reading was taken 6 min after the initial mixing. Trolox (0.1 mg/mL) and Quercetin (0.05 mg/mL) were used as positive controls. The activities of the samples were evaluated by comparison with a control (containing 4.85 mL of ABTS solution and 0.15 mL of 45% ethanol). Each sample was measured in triplicate and averaged. This activity is given as percentage ABTS ^+^ scavenging that is calculated by the following formula:

$$ABTS^{+}\;scavenging\;activity\;(\%)\;=\;\left[\left(A_{c}–A_{s}\right)/A_{c}\right]\;\times \;100$$

where *A*_C_ is the absorbance value of the control and *A*_S_ is the absorbance value of the added samples test solution.

### Evaluation of total antioxidant activity by phosphomolybdenum method

The total antioxidant capacity of the extracts was evaluated according to the method described by Prieto *et al*.[20] An aliquot of 0.5 mL of samples solution was combined with 4.5 mL of reagent solution (0.6 M sulfuric acid, 28 mM sodium phosphate, and 4 mM ammonium molybdate). In case of blank, 0.5 mL of 45% ethanol was used in place of sample. The tubes were incubated in a boiling water bath at 95°C for 90 min. After the samples were cooled to room temperature, the absorbance of the aqueous solution of each sample was measured at 695 nm against blank in UV-2450 spectrophotometer (Shimadzu, Japan). The total antioxidant activity was expressed as the absorbance of the sample at 695 nm. The higher absorbance value indicated higher antioxidant activity.[21]

### Statistical analysis

Results were given as mean ± standard deviation of 3 replicates. Experimental results were analyzed by SPSS version 16.0 (SPSS Inc. Chicago, IL). Differences between means were determined using one-way ANOVA and Duncan’s test. The level of statistical significance was set at *P* ≤ 0.05.

## RESULTS

The effect of extraction temperature on total phenolic and total flavonoid content

Table 1 shows the effect of different extraction temperatures on TPC and TFC of the extracts obtained. Increasing the extraction temperature increased TPC and TFC. However, at temperatures 90°C and above, the values were not significantly different (*P* > 0.05) with increasing extraction temperature. Although the maximum amount of TPC and TFC was obtained during extraction at 100°C, the values were not significantly different (*P* > 0.05) from those obtained at 90°C. For the extraction temperatures studied, temperature below 80°C showed a significantly lower total phenolic and total flavonoid contents compared with extractions at 90°C and 100°C. TPC and TFC of all the extracts at different temperatures studied increased in the order: 100°C > 90°C > 80°C > 60°C > 40°C.

**Table 1:** Effect of extraction temperature on total phenolic content and total flavonoid content

Extraction temperature (°C)	TPC (mg GA/g dry material)	TFC (mg Kaempferol/g dry material)
40	13.95 ± 0.19^a^	19.16 ± 0.11^a^
60	21.28 ± 0.37^b^	27.04 ± 0.11^b^
80	27.26 ± 0.7^c^	35.76 ± 0.11^c^
90	34.30 ± 0.50^d^	44.14 ± 0.22^d^
100	36.68 ± 0.62^d^	47.52 ± 0.21^d^

TPC: Total phenolic content, TFC: Total flavonoid content, GA: Gallic acid, Values are means of 3 replicates ± SD, different letters within the same column indicate significant difference at *P* < 0.05 by Duncan’s test

### The effect of extraction temperature on free radical-scavenging capacity

The results of free radical-scavenging capacity of the extracts obtained at different temperatures by DPPH and ABTS methods are shown in Table 2. All of the extracts demonstrated inhibitory activity against both the DPPH free radical and the ABTS cation free radical. The order of free radical-scavenging capacity of the extracts was 100°C > 90°C > 80°C > 60°C > 40°C. At 100°C, the extracts showed the highest free radical-scavenging capacity; however, the values were not significantly different from those (*P* > 0.05) obtained at 90°C and those of standard materials.

**Table 2:** Effect of extraction temperature on free radical-scavenging capacity

Extraction temperature (°C)	DPPH (%)	ABTS (%)
40	46.10 ± 2.36_a_	28.65 ± 2.04_a_
60	67.35 ± 3.17_b_	38.74 ± 1.50_b_
80	83.67 ± 2.50_c_	50.10 ± 0.30_c_
90	88.69 ± 0.50_d_	62.91 ± 1.87_d_
100	89.67 ± 0.06_d_	68.27 ± 1.36_d_
Trolox	91.69 ± 0.28_d_	67.75 ± 2.88_d_
Quercetion	90.19 ± 0.17_d_	63.62 ± 1.23_d_

DPPH: 1,1-diphenyl-2-picrylhydrazyl, ABTS: 2,2-azino-bis-(3-ethylbenzthiazoline-6-sulfonic acid, Values are means of 3 replicates ± SD, different letters within the same column indicate significant difference at *P* < 0.05 by Duncan’s test

### The effect of extraction temperature on total antioxidant activity

**Figure 1:**
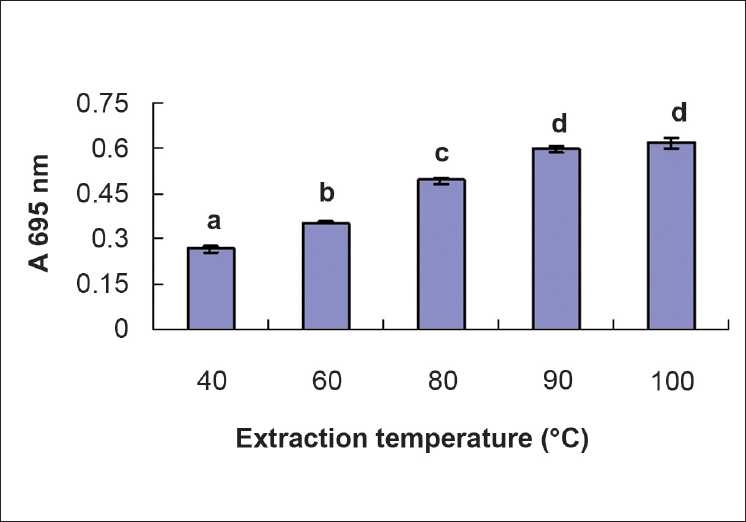
Effect of extraction temperature on total antioxidant activity. Values are means of 3 replicates ± SD; different letters within the same column indicate significant difference at *P* < 0.05 by Duncan’s test

The phosphomolybdate method has been used routinely to evaluate the total antioxidant capacity of plant extracts.[20,21] In the presence of extracts, Mo (VI) is reduced to Mo (V) and forms a green colored phosphomolybdenum V complex, which shows a maximum absorbance at 695 nm. Figure 1 shows that the antioxidant capacity of the extracts of *G. divaricata* leaves at different temperatures can be ranked in the order 100°C > 90°C > 80°C > 60°C > 40°C. Extracts at 100°C showed the highest total antioxidant activity; however, the values were not significantly different from those (*P* > 0.05) obtained at 90°C. This implies that the extracts at 90°C and 100°C have a strong antioxidant ability (*P* < 0.05) as compared with the extracts obtained at lower temperatures.

### The correlation between TPC and antioxidant activity

The correlation between TPC and TFC, antioxidant activity, and free radical-scavenging capacity of the extracts obtained at different temperatures is interesting to note. The Pearson correlation analysis [Table 3] revealed that the TPC and TFC showed strong correlations with DPPH radical scavenging capacity, ABTS cation radical scavenging capacity, and total antioxidant activity, with the correlation coefficient (*R*^2^) 0.9229, 0.9952, and 0.9872, respectively. This indicates that the antioxidant activity of the extract from *G. divaricata* leaves is due to its phenolic constituents. These results are in accordance with other reports in the literature, which showed positive strong correlation between antioxidant activities and TPCs.[22] Phytochemical screening showed that kaempferol and its derivatives are the major bioactive components in *G. divaricata* leaves, and this is in agreement with the study by Chen *et al*.,[9] and these phytochemicals have been reported to possess high antioxidant and hypoglycemic activity.[23,24]

**Table 3:** Correlations between the antioxidant activities and TPC and TFC of extracts of Gynura divaricata leaf

Antioxidant assays	Correlation *R*_2_	
	TPC	TFC
DPPH radical scavenging ability	0.9229	0.9145
ABTS radical scavenging ability	0.9951	0.9980
Total antioxidant activity	0.9872	0.9930

TPC: Total phenolic content, TFC: Total flavonoid content, DPPH: 1,1-diphenyl-2-picrylhydrazyl, ABTS: 2,2-azino-bis-(3-ethylbenzthiazoline-6-sulfonic acid

## DISCUSSION

**Figure 2:**
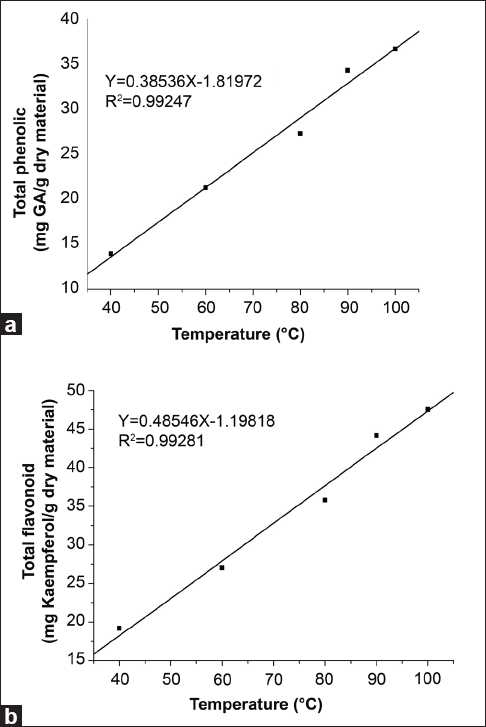
Relationship between (a) extraction temperature and total phenolic contents, (b) extraction temperature and total flavonoid contents

The present study was focused on TPC and TFC, in addition to the antioxidant activity of *G. divaricata* leaf extracts obtained at different temperatures. The influence of extraction temperature on TPC, TFC, and antioxidant activity of the 45% ethanol extracts was investigated. For all the extraction temperatures surveyed, the highest TPC, TFC, and antioxidant activities were observed in the extracts obtained at 100°C followed by extraction at 90°C. Significant lower levels of TPC and TFC were demonstrated by extractions at temperatures below 80°C. The TPC and TFC of extracts have strong positive correlation with the extraction temperature, with the correlation coefficient (*R*^2^) 0.9925 and 0.9928, respectively [Figure 2]. The results of the present study also indicate that there was a significant reduction in the total antioxidant activity and free radical-scavenging capacity of the extracts obtained at temperatures below 80°C compared to those extracted at 90°C or 100°C. This was expected due to the elevation the TPC and TFC of extracts at higher temperatures. Our findings are in contrast to Akowuah *et al*.’s study[13] on *G. procumbens*, indicating that TPC and free radical scavenging activities decreased with increasing extraction temperatures. Also our findings implied that extraction temperatures will have different effects on the bioactive components of identical genus plants, although they may share some uniform constituents. It is generally considered that extraction at a high temperature will lead to a degradation of some bioactive compounds, whereas recently some researches have demonstrated that this is not always true, especially with regard to the polyphenols with antioxidant activity. Increasing the extraction temperature has been found to enhance the recovery of phenolic compounds as described in previous reports.[25,26] The mechanism maybe is that increasing extraction temperature promotes solvent extraction by enhancing both diffusion coefficients and the solubility of polyphenol content.[27,28] Also, increasing extraction temperature will contribute to the release of bound polyphenols in plants with the breakdown of cellular constituents of plant cells which leads to increased cell membrane permeability. Moreover, release of these bound polyphenols could further reduce the chances of those polyphenols coagulating with lipoprotein. Thereby enhancing solubility of the polyphenols and inhibiting coagulation with lipoprotein will increase polyphenols yield.[29]

As a dual purpose plant, for its medicinal and edible purpose, *G. divaricata* was extensively used to treat diabetics in folk medicine. Flavonoids are the bioactive constituents of the plant and are influenced by the decoction temperature; however, there is not enough information about decoction temperature of this medicinal plant. We have herein provided the available information about decoction temperature of the plant; demonstrated the antioxidant activities of the ethanol extract of *G. divaricata*; and confirmed the significant influence of extraction temperature on the phenolic and flavonoid contents. The results emphasize the extract of *G. divaricata* has possessed significant antioxidant properties. Therefore, *G. divaricata* would be a potential source of natural antioxidants and nourishment. The consumption of *G. divaricata* in the local people might give positive function of health protection against oxidative damages. With the ascertained antioxidant activity of this plant, the separation and identification of the antioxidative components in the ethanol extract and using response–surface methodology (RSM) optimization, the extraction of phenolic compounds should be further investigated.

## CONCLUSION

The optimal extraction temperature of *G. divaricata* leaf, chosen as a comparison between the yield of phenolic components (TPC and TFC) and their antioxidant and free radical-scavenging capacities (DPPH and ABTS), were 45% ethanol for 30 min at 90°C. Significant correlation was found for TPC and TFC with different extraction temperatures. Also TPC and TFC have significant correlation with the total antioxidant activity and free radical-scavenging capacities. This study provides constructive information for further optimization on the extraction of phenolic compounds from *G. divaricata* using RSM.
